# Recurrent network for multisensory integration-identification of common sources of audiovisual stimuli

**DOI:** 10.3389/fncom.2013.00101

**Published:** 2013-07-25

**Authors:** Itsuki Yamashita, Kentaro Katahira, Yasuhiko Igarashi, Kazuo Okanoya, Masato Okada

**Affiliations:** ^1^Graduate School of Frontier Sciences, The University of TokyoKashiwa, Chiba, Japan; ^2^Graduate School of Arts and Sciences, The University of TokyoMeguro, Tokyo, Japan; ^3^RIKEN Brain Science InstituteWako, Saitama, Japan; ^4^ERATO, Okanoya Emotional Information Project, Japan Science Technology AgencyWako, Saitama, Japan

**Keywords:** causality inference, multisensory integration, spatial orientation, recurrent neural network, Mexican-hat type interaction

## Abstract

We perceive our surrounding environment by using different sense organs. However, it is not clear how the brain estimates information from our surroundings from the multisensory stimuli it receives. While Bayesian inference provides a normative account of the computational principle at work in the brain, it does not provide information on how the nervous system actually implements the computation. To provide an insight into how the neural dynamics are related to multisensory integration, we constructed a recurrent network model that can implement computations related to multisensory integration. Our model not only extracts information from noisy neural activity patterns, it also estimates a causal structure; i.e., it can infer whether the different stimuli came from the same source or different sources. We show that our model can reproduce the results of psychophysical experiments on spatial unity and localization bias which indicate that a shift occurs in the perceived position of a stimulus through the effect of another simultaneous stimulus. The experimental data have been reproduced in previous studies using Bayesian models. By comparing the Bayesian model and our neural network model, we investigated how the Bayesian prior is represented in neural circuits.

## 1. Introduction

We are surrounded by many sources of sensory stimulation, i.e., many sights and sounds. Moreover, we can recognize who is speaking in a conversation even when there are many people and sounds around us. To perform such recognition, we have to integrate correct pairs of stimuli; the movements of a person's mouth and the sound of his/her voice. Thus, it is important to determine how we judge which pairs of audiovisual stimuli are related and how we integrate related cues. That is, we must study multisensory integration in order to elucidate how our brains link multiple sources of information. There is a good example of audiovisual integration known as the ventriloquism effect in which the perceived location of a ventriloquist's voice is altered through the movement of a dummy's mouth (Howard and Templeton, 1966). It is also known that the ventriloquism effect can be elicited under artificial experimental conditions such as a spot of light or a beep (Bertelson and Aschersleben, 1998; Pavani et al., 2000; Lewald et al., 2001; Hairston et al., 2003; Alais and Burr, 2004; Wallace et al., 2004). Several theoretical models based on Bayesian inference have been proposed to explain the data from psychophysical experiments on the ventriloquism effect (Körding et al., 2007; Sato et al., 2007). Although Bayesian inference gives a normative account as to the computational principle, it does not indicate how the nervous systems actually implement the computation.

To provide insights into the neuron dynamics related to sensory integration, several studies have constructed neural network models that implement Bayesian inference (Pouget et al., 1998; Ma et al., 2006). When stimuli have a common cause, their models are able to extract encoded information from the activities of large populations of neurons as reliably as the maximum likelihood is able to do (Deneve et al., 1999; Latham et al., 2003). However, when stimuli have distinct sources, the models cannot work correctly because they bind cues even when the stimuli do not have the same source. When the stimuli have distinct causes, the brain has to estimate the causal structure of the stimuli and extract information separately from each stimuli. We constructed a recurrent network model that can implement computations related to multisensory integration by changing the method of divisive normalization in the model of Deneve et al. (1999). We found that our model could estimate not only the locations of the sources of the stimuli but also the number of sources. By using computer simulation, we showed that the model accounts for the data of psychophysical experiments that have been explained by the Bayesian model. To elucidate how our brains implement a Bayesian prior distribution, we tried to determine which neural connectivities represent the prior distribution.

## 2. Model

We constructed a single layer recurrent network consisting of *N* = 1000 analog neurons with identical spatial receptive fields. Here, we will label a neuron, *i*, by an angle θ_*i*_ and express the firing rate as a function of θ; therefore, a neural state, *u*_*i*_, describes the firing rate of the neuron population (including both excitatory and inhibitory neurons) with the preferred angle, *i*. In order to reduce the number of parameters and facilitate analysis of the system's behavior, we will study a simpler model, in which the excitatory and inhibitory populations are collapsed into a single equivalent population. To model a cortical hypercolumn consisting of a single layer of neurons, we assumed that the preferred orientations are evenly distributed from −50 to 50 deg and divided 100 deg into *N* = 1000 sections, that is, θ_*i*_ = 0.1 × *i* − 50 deg. The neural state, *u*_*i*_, is determined by inputs, *a*_*i*_, as

(1) $$a_{i}(t)=h_{i}+\sum_{j}^{N}J_{ij}u_{j}(t),$$

where *h*_*i*_ represents an external input and the second term of the right-hand side of the equation represents a recurrent input. Using *a*_*i*_, we defined the firing rate *u*_*i*_ as

(2) $$u_{i}(t+1)=\frac{{\left[a_{i}(t)\right]}_{+}}{1+\frac{1}{N}\sum_{j}^{N}{\left[a_{j}(t)\right]}_{+}}.$$

To keep *u*_*i*_ positive, we used the threshold linear function [*a*_*i*_]_+_ ([*a*_*i*_]_+_ = *a*_*i*_ if *a*_*i*_ > 0, [*a*_*i*_]_+_ = 0 if *a*_*i*_ ≤ 0). To control the gain of the firing rate *u*_*i*_, we used divisive normalization Carandini et al. (1997). The interaction in the network turns noisy input into a smooth hill shape. The cap coordinate of the hill gives an estimate of the orientation. In a previous study, Deneve et al. (1999) defined a function *u*_*i*_(*t*) in terms of the square of the input *a*_*i*_

(3) $$\hat{u}(t)=\frac{a_{i}{\left(t\right)}^{2}}{1+\frac{1}{N}\sum_{j}^{N}a_{j}{\left(t\right)}^{2}}.$$

**Figure 1:**
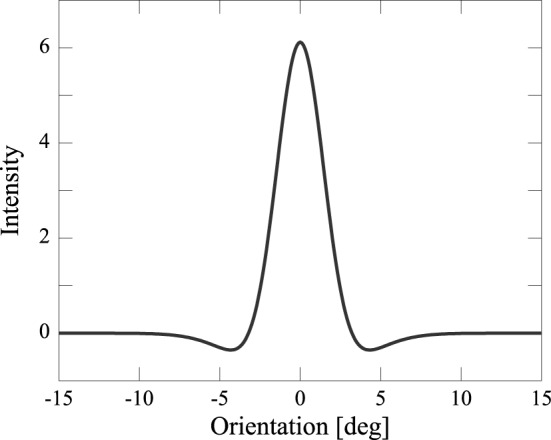
**Mexican-hat-type connectivity *J*_*ij*_: excitations are given to nearby neurons, inhibitions to distant neurons**.

In order to collapse the excitatory and the inhibitory populations into a single equivalent population, we assumed that the synaptic weight, *J*_*ij*_, is a Mexican-hat-type connectivity: excitations are given to nearby neurons, inhibitions to distant neurons (Figure 1; Amari, 1977; Shadlen et al., 1996). We defined:

(4) $$J_{ij}=\frac{M_{1}}{\sqrt{2\pi \sigma_{1}^{2}}}\exp\left(\frac{-{\left(\theta_{i}-\theta_{j}\right)}^{2}}{2\sigma_{1}^{2}}\right)-\frac{M_{2}}{\sqrt{2\pi \sigma_{2}^{2}}}\exp\left(\frac{-{\left(\theta_{i}-\theta_{j}\right)}^{2}}{2\sigma_{2}^{2}}\right).$$

The parameters σ_1_, σ_2_, respectively define the range of the excitatory connection and lateral inhibition. Here, we set *M*_1_ = 28, *M*_2_ = 10, σ_1_ = 1.5 [*deg*], and σ_2_ = 3 [*deg*]. The two features in our model, i.e., weak normalization and lateral inhibition, make differences between ours and Deneve's model, and they enable our model to reproduce the results of psychophysical experiments (as discussed in the Results).

**Figure 2:**
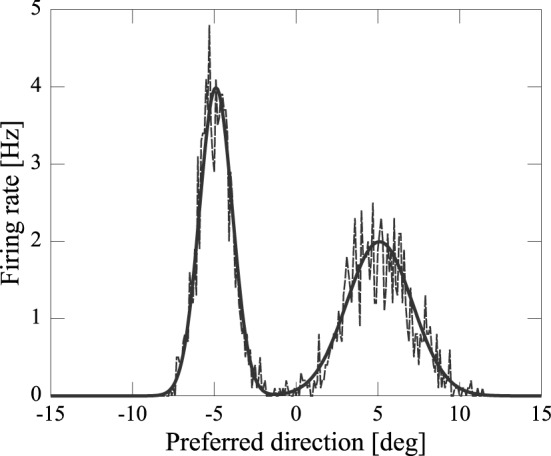
**Example of input to the network (broken line) and mean input (solid line)**.

Let us consider an external input, *h*, from either a preceding layer or from the external world. The external input of neuron *i*, *h*_*i*_, is dependent on the orientation encoded in the previous layer and is Gaussian distributed with mean 〈*h_i_*〉 and variance σ^2^_*i*_. We define

(5) $$h_{i}\text{​}=\text{​}\frac{M_{V}}{\sqrt{2\pi \sigma_{V}^{2}}}\exp\left(-\frac{\text{​}{\left(\theta_{i}-x_{V}\right)}^{2}}{2\sigma_{V}^{2}}\right)\text{​ }+\;\frac{M_{A}}{\sqrt{2\pi \sigma_{A}^{2}}}\exp\text{​}\left(-\frac{{\left(\theta_{i}-x_{A}\right)}^{2}}{2\sigma_{A}^{2}}\right)\text{​}+\text{​}z_{i},\text{​}$$

where *z*_*i*_ denotes noise. We set σ^2^_*i*_ to the mean activity, i.e., σ^2^_*i*_ = 〈*h*_*i*_〉, which better approximates the noise measured in the cortex Shadlen and Newsome (1994). The standard deviations σ_*V*_ and σ_*A*_ respectively represent the uncertainties of the visual and audio input. Note that the strength of the input activity, $\frac{M}{\sqrt{2\pi \sigma^{2}}}$, is determined not only by *M* but also by the uncertainty of the input, σ, in our model. We assumed that the visual input is more reliable than the audio input. To investigate the effect of the difference in uncertainty between visual and audio input, we fixed *M*_*V*_ = *M*_*A*_ = 10, σ_*V*_ = 1 [*deg*], and σ_*A*_ = 2 [*deg*]. Thus, the input strength of visual input is larger than that of audio input, i.e., $\frac{M_{V}}{\sqrt{2\pi \sigma_{V}^{2}}}>\frac{M_{A}}{\sqrt{2\pi \sigma_{A}^{2}}}$. An example of external input to the network is given in Figure 2.

**Figure 3:**
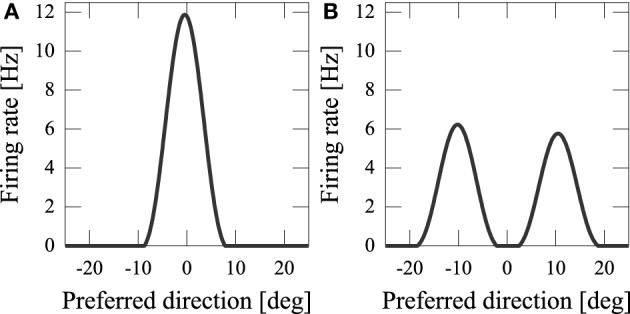
**Output of network model. (A)** Common cause; **(B)** independent cause.

Now let us explain *x*_*V*_ and *x*_*A*_ in Equation 5. *x*_*V*_ and *x*_*A*_ represent the input locations of audiovisual stimuli. We assume that the audio and visual stimuli are Gaussian distributed: 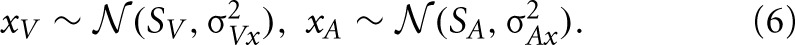


We fix σ_*Vx*_ = 3 [deg] and σ_*Ax*_ = 6.5 [deg]. Here, 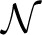
(μ, σ_*p*_) means a Gaussian distribution with mean μ and standard deviation σ. The external input, *h*_*i*_, is given in the initial five steps (0 ≤ *t* ≤ 4). The noisy input, *h*_*i*_, determines the initial state of *u*_*i*_ [*a*_*i*_(0) = *h*_*i*_]. Because of the recurrent connections and neural dynamics (See Equation 1 and Equation 2), the noisy neural states become a smooth hill whose peak indicates the estimated position of the audiovisual stimuli (Figure 3).

## 3. Results

**Figure 4:**
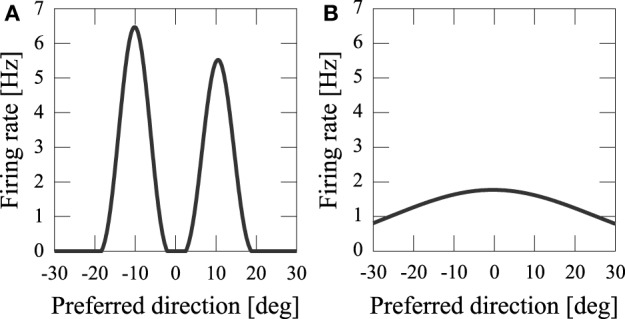
**Model comparison in the case of independent cause. (A)** Our network model; (**B)** weak normalization model without lateral inhibition.

By using computer simulations, we showed that our network model can estimate the position(s) of the sources of audiovisual stimuli with a disparity between the stimuli. We found that while previous models could not reproduce psychophysical experiments of the audiovisual integration, our results are consistent with both experimental observations and Bayesian inference. If the disparity of the input stimuli was small (*x*_*A*_ − *x*_*V*_ = 5 [deg]), the stimuli were integrated with a high rate (about 70%) (Figure 3A). If the disparity was large, they were estimated as distinct stimuli (Figure 3B), something which could not be reproduced in previous models where the normalization term in Equation 2 is determined by the square sum Deneve et al. (1999). We found that in the previous network model, they were estimated not as distinct stimuli but as a united stimulus for any spatial disparity. The failure of Deneve's model to reproduce the phenomenon is partly the result of the strong divisive normalization they used (Equation 2), because the strong divisive normalization prunes weak multiple input peaks and extracts the maximum peak. Another reason for the failure of reproduction is the lateral inhibition between neurons. Figure 4 compares the models in the case of independent causes. Similarly to the Deneve's model, in a weak normalization model without lateral inhibition, they were estimated not as distinct stimuli but as a united stimulus for any spatial disparity, as shown in Figure 4B Marti et al. (2013). Thus, both weak normalization and lateral inhibition in our model are important for reproducing the results of the psychophysical experiments on audiovisual integration.

### 3.1. Effect of sensory noise

**Figure 5:**
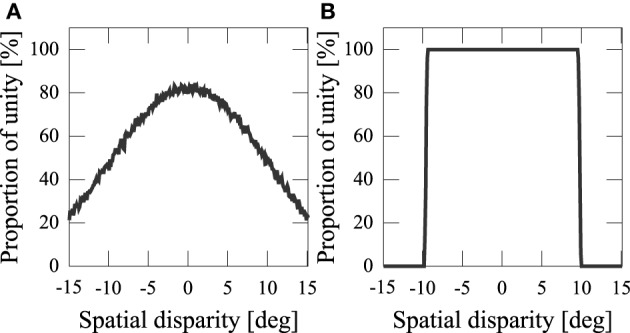
**Frequency of the network model estimating stimuli having a common cause. (A)** Proportion of unity with sensory noise [*x*_*V*_ ~ 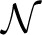
(*S*_*V*_, σ^2^_*Vx*_), *x*_*A*_ ~ 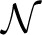
(*S*_*A*_, σ^2^_*Ax*_)]. **(B)** Proportion of unity without noise (*S*_*V*_ = *x*_*V*_, *S*_*A*_ = *x*_*A*_).

We assumed that information about the orientations of the audiovisual stimuli from sense organs, *x*_*V*_, *x*_*A*_, are corrupted with sensory noise. This noise makes the output probabilistic (Figure 5A). If we didn't add noise, the number of sources would be completely determined by the spatial disparity *D* (Figure 5B). Experiments have shown that people estimate the number of sources stochastically Wallace et al. (2004).

### 3.2. Bias

Psychophysical experimental research has reported that when audiovisual stimuli were estimated as distinct stimuli, the estimated position of the auditory stimuli was away from the actual position of the auditory input Wallace et al. (2004). To examine how the perception of common versus distinct causes affects the estimation of the auditory stimuli position, *Ŝ*_*A*_, we calculated the localization bias,

(7) $$bias=\frac{{\hat{S}}_{A}-S_{A}}{S_{V}-S_{A}}.$$

**Figure 6:**
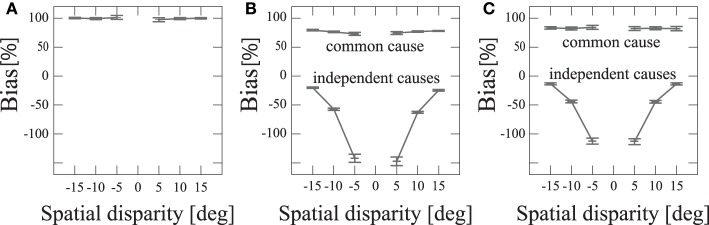
**Localization bias with spatial disparity *D***. Error bars represent SEMs. **(A)** Previous model Deneve et al. (1999). **(B)** Proposed model. **(C)** Bayesian model Körding et al. (2007); Sato et al. (2007). “Common cause” refers to the situation in which the model regarded that two sensory signals have a common cause (i.e., the network converged to a single bump state) and “independent cause” refers to the situation in which the network regarded that two sensory signals have independent causes (i.e., the network converged to a state of two single bumps or the MAP estimate of the Bayesian model corresponds to two sources). The negative bias indicates that the perceived auditory position is on the opposite side of the true position with respect to the position of the visual stimulus.

We performed 500 simulations and averaged the localization bias for each disparity between the audiovisual stimuli and for each case, i.e., common and distinct. We compared our model and the previous model of Deneve et al. (1999). In the previous model, the stimuli were unified with any spatial disparity (Figure 6A). The value of the localization bias was nearly 100% with all spatial disparities. This means their model estimated the audio stimulus as noise. Our model made estimates about whether stimuli have a common cause or distinct causes stochastically (Figure 6B). When two stimuli were unified, the localization bias was nearly 80%. This indicates that when there was a common cause, the estimated auditory stimulus would be at a position that was on average very close to that of the visual stimulus. On the other hand, in the case of distinct causes, the localization bias took a negative value and was increasingly negative for smaller disparities. These results indicate that the estimated auditory position seems to be pushed away from the location of the visual stimulus, as was experimentally observed Wallace et al. (2004).

## 4. Bayesian prior in a neural network

Bayesian inference is a method of reasoning that combines prior knowledge about the world with current input data. To be more precise, from experience we may learn how likely two co-occurring signals (visual and auditory signals) are to have a common cause versus two independent causes. Using the Bayesian prior, a Bayesian inference model integrates those pieces of information to estimate if there is a common cause and to estimate the positions of cues. Previous studies have reported that Bayesian inference could explain the pattern of localization bias as reproduced by our model (Figure 6C) (Körding et al., 2007; Sato et al., 2007). Considering that our neural network model and the Bayesian model could explain the same psychophysical experiment, there should be a neural connection in our model that represents prior information. We searched for the parameter of our network model that corresponded to the prior information of the likelihood of sensory integration.

### 4.1. Multisensory integration in the neural network

To simplify the comparison between the network model and Bayes model, let us consider a case in which we receive sensory inputs without noise (*x*_*V*_ = *S*_*V*_, *x*_*A*_ = *S*_*A*_). The distance between audiovisual stimuli *D* determines the causal structure in this case (Figure 5B), and we can determine the integration threshold *D*^*Net*^_0_ (the distance within which the auditory and visual signals are integrated). When *D*^*Net*^_0_ is determined, we can calculate the proportion of integration with noise as follows. When the distance between the audiovisual inputs, *x*_*V*_ − *x*_*A*_ = *D*_*input*_, is lower than *D*^*Net*^_0_, stimuli integrate. *D*_*input*_ is drawn from a normal distribution with mean *S*_*V*_ − *S*_*A*_ = *D*, which is the distance between the original positions of the audiovisual stimuli, and standard deviation $\sqrt{\sigma_{Vx}^{2}+\sigma_{Ax}^{2}}$, which is the sum of the auditory and visual noise. Using *D*^*Net*^, we obtain the proportion of integration as a function of *D*,

$$I(D)=\int_{D-D_{0}^{Net}}^{D+D_{0}^{Net}}\frac{1}{\sqrt{2\pi (\sigma_{Vx}^{2}+\sigma_{Ax}^{2})}}\exp\left(-\frac{t^{2}}{2(\sigma_{Vx}^{2}+\sigma_{Ax}^{2})}\right)dt.$$

*D*^*Net*^_0_ determines the likelihood of sensory integration. We investigated the relationship between the parameters of the neural connection *J*_*ij*_ and the Bayesian prior distribution regarding the integration threshold.

### 4.2. Integration threshold in bayesian model

Using the Bayesian approach, we can also calculate the integration threshold *D*^*Bay*^_0_ (distance within which auditory and visual signals are integrated in the Bayesian view) as follows (Körding et al., 2007). We determine whether the stimuli originate from the same source (*C* = 1) or two sources (*C* = 2). The perceived locations of audiovisual stimuli *x*_*V*_, *x*_*A*_ are shifted from their original position using Gaussian noise with standard deviations of σ_*V*_, σ_*A*_. Accordingly, we calculate the probability of *C* = 1 using Bayes' theorem (Körding et al., 2007):

$$\begin{array}{l} p(C=1|x_{V},x_{A})\\ \;=\frac{p(x_{V},x_{A}|C=1)p_{co}}{p(x_{V},x_{A})}\;(p(C=1)\equiv p_{co})\\ \;=\frac{p(x_{V},x_{A}|C=1)p_{co}}{p(x_{V},x_{A}|C=1)p_{co}+p(x_{V},x_{A}|C=2)\left(1-p_{co}\right)}. \end{array}$$

When the source locations from the audiovisual signals are uniformly distributed in the spatial range [−*a*/2, *a*/2], we obtain

(8) $$p(C=1|x_{V},x_{A})=\frac{aq(D)p_{co}}{aq(D)p_{co}+1-p_{co}}$$

where

(9) $$q(D)\equiv \frac{1}{\sqrt{2\pi (\sigma_{V}^{2}+\sigma_{A}^{2})}}\exp\left\{-\frac{D^{2}}{2(\sigma_{V}^{2}+\sigma_{A}^{2})}\right\}.$$

**Figure 7:**
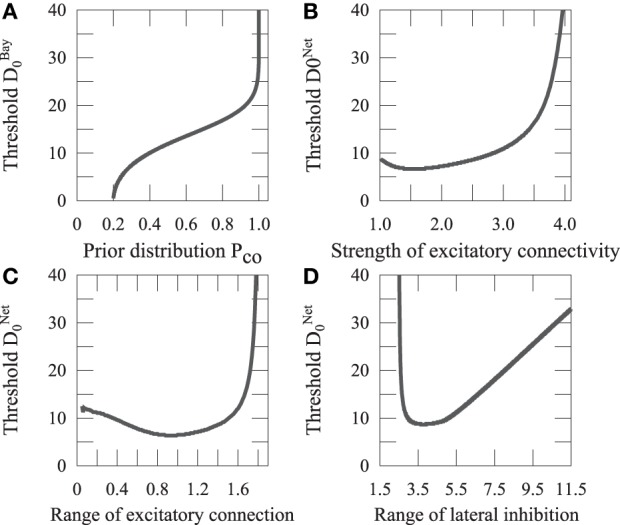
**Threshold of integration. (A)** The thresholds of the Bayesian model are plotted with the prior of sensory integration. **(B)** The thresholds of the network model are plotted showing the ratio of the strengths of the excitatory connection, *M*_1_, and inhibitory connection, *M*_2_, of the recurrent network. **(C)** The thresholds of the network model are plotted with the range of excitatory connection, σ_1_. **(D)** The thresholds of the network model are plotted with the range of lateral inhibition, σ_2_.

We assume that the Bayesian model reports the same source when *p*(*C* = 1|*x*_*V*_, *x*_*A*_) > *p*(*C* = 2|*x*_*V*_, *x*_*A*_). We define *D*^*Bay*^_0_ as a distance *D* that satisfies *p*(*C* = 1|*x*_*V*_, *x*_*A*_) = *p*(*C* = 2|*x*_*V*_, *x*_*A*_). As shown in Equation 8, the Bayesian prior *P*_*co*_ affects the judgment of unity. We investigated how *P*_*co*_ affects *D*^*Bay*^_0_ (Figure 7).

When the causal structure is defined, we can calculate the optimal estimate of the stimulus position for the cases of *C* = 1 and *C* = 2. When the audiovisual stimuli have independent causes, the optimal solutions are

(10) $${\hat{x}}_{V,C=2}=x_{V},\;{\hat{x}}_{A,C=2}=x_{A}.$$

When the audiovisual stimuli have a common cause, the optimal solution is

(11) $${\hat{x}}_{V,C=1}={\hat{x}}_{A,C=1}=\frac{\frac{x_{V}}{\sigma_{V}^{2}}+\frac{x_{A}}{\sigma_{A}^{2}}}{\frac{1}{\sigma_{V}^{2}}+\frac{1}{\sigma_{A}^{2}}}.$$

We calculated the localization bias using the Bayesian model (Figure 6C). Here, we fixed *P*_*co*_ = 0.2, σ_*V*_ = 3 [deg], and σ_*A*_ = 6.5 [deg].

Both the Bayesian prior *P*_*co*_ and recurrent connectivity *J*_*ij*_ affect the integration threshold *D*_0_. Thus, the integration threshold *D*_0_ validates the idea that the Bayesian prior *P*_*co*_ corresponds to a recurrent connectivity *J*_*ij*_ in the cortical neural network.

### 4.3. Network connectivity represents bayesian prior *P*_*co*_

Synaptic plasticity is thought to be the basic phenomena underlying learning. It could be said that a neural network learns a Bayesian prior by changing its connectivity. We investigated how the parameters of the network connectivity *J*_*ij*_ affect the integration threshold, as shown in Figure 7. *D*^*Net*^_0_ increases with the ratio between the strength of excitatory connection, *M*_1_, and that of the inhibitory connection, *M*_2_. It approximately increases with the range of excitatory connection, σ_1_, similarly to the ratio between *M*_1_ and *M*_2_, whereas it varies with a non-monotonic shape for the range of lateral inhibition, σ_2_, as illustrated in Figure 7D.

Let us focus on the excitatory connection that could be changed through Hebb's learning rule. As shown in Figures 7A, B, *D*^*Net*^_0_ increases with *M*_1_ in the same way as *D*^*Bay*^_0_ increases with *P*_*co*_. This means that the Bayesian prior *P*_*co*_ is represented as *M*_1_ in the network model. This result suggests that the neural network achieves Bayesian inference through learning appropriate prior information by adjusting the excitatory connection *M*_1_.

## 5. Discussion

We constructed a recurrent network model that distinguishes whether or not audiovisual stimuli have a common cause or distinct causes. We showed that our model not only estimates the number of sources, but also reproduces the localization bias, as observed in psychophysical experiments Wallace et al. (2004). Previous studies have revealed that the Bayesian ideal observer model could explain psychophysical data on sensory integration Körding et al. (2007); Sato et al. (2007). Although a Bayesian model gives a normative account of the computational principle, it does not provide a neural implementation of optimal causal inference. Our model is a biologically plausible one of cortical circuitry, and it provides information about how the nervous system can implement the computation Carandini et al. (1997). To reveal how the nervous system implements Bayes' inference, we investigated the relationship between the synaptic connection of the proposed model and the prior distribution in the Bayesian model. We found that the strength of the excitatory connection represents the prior distribution for the probability of integration.

Previous research has used divisive normalization for the firing rate, serving as a gain control. The network model extracted variables encoded by a population of noisy neurons Deneve et al. (1999). The neural activities converged to a smooth stable peak, and the position of the peak depended on the variables. Therefore, the position could be used to estimate these quantities in their model. Moreover, through proper tuning of the parameters, the model closely approximated the maximum likelihood, which would be used by an ideal observer in most cases of interest. However, two or more localized activities could not coexist in the previous network model. The model thus could not simultaneously estimate information about multiple sources, which is needed for living in a natural environment. We found that strong divisive normalization makes it hard for localized activities to coexist. Iteration of Equation 3 makes the ratio of local excitations large, and eventually, only the largest one can survive. This effect occurs if the exponent is greater than 1. We constructed a model in which an arbitrary number of local excitations could coexist by making the exponent equal to one. This simple normalization can be biologically implemented in a linear computation and shunting inhibition Carandini et al. (1997). Although our network may not achieve optimal inference for each source position, it is biologically plausible and can reproduce the properties of auditory-visual integration observed in psychophysical experiments. These results imply that normalization with a threshold linear function is important in multisensory integration with causal inference.

We reproduced the results of psychophysical experiments showing localization bias in audiovisual integration. Whenever stimuli were unified, the model estimated that the auditory position would shift to the location of the visual stimulus. This phenomenon is caused by the difference in the reliability of the stimuli. That is, because visual information for source localization is much more reliable than auditory information, vision dominates sound. Moreover, it is also known that when an auditory signal is more reliable than a visual signal, sound dominates vision Alais and Burr (2004). It is reported that localization bias is observed in some cross-modal cues Pavani et al. (2000). Our model represents the reliability of stimuli by the strength of the input activity. It can be generalized to other types of cue integration by changing the strength of the input activity.

The results of psychophysical experiments have been explained using Bayes' inference Körding et al. (2007); Sato et al. (2007). Bayes' inference is a method of reasoning that combines prior knowledge with current input data. In our brains, information about the external world is estimated on the basis of prior knowledge Doya et al. (2007). However, until now, it was unknown how prior knowledge can be represented in a neural circuit. We investigated how a neural network can implement prior knowledge. Our results suggest that neural networks learn an appropriate prior with synaptic plasticity.

In the Bayesian model, negative bias is assumed to be caused by sensory noise Körding et al. (2007); Sato et al. (2007). Stimuli are unified when the distance between the perceived locations of audiovisual stimuli which are shifted from their original positions is smaller than *D*^*Bay*^_0_; on the other hand, when it is larger than *D*^*Bay*^_0_, the stimuli are not unified. The averaged bias of the non-unified case takes on a negative value. In our neural network model, not only sensory noise but also the interaction of localized activities has an effect on the negative bias. Localized activities repel each other through the effect of a Mexican-hat type of connectivity (Figure 3). This corresponds to implementing the prior distribution such that of the likely positions of different input sources, which has not been implemented in the previous Bayesian models Körding et al. (2007); Sato et al. (2007). It is unclear where causal inference is performed in the brain. If the repulsive effect were to be observed in a brain region that performs multisensory integration, it would support the notion that our model is actually implemented in the brain.

### Conflict of interest statement

The authors declare that the research was conducted in the absence of any commercial or financial relationships that could be construed as a potential conflict of interest.

## References

[B1] Alais D. Burr D. (2004). The ventriloquist effect results from near-optimal bimodal integration. Curr. Biol. 14, 257–262 10.1016/j.cub.2004.01.029 14761661

[B2] Amari S. (1977). Dynamics of pattern formation in lateral-inhibition type neural fields. Biol. Cybern. 27, 77–87 10.1007/BF00337259 911931

[B3] Bertelson P. Aschersleben G. (1998). Automatic visual bias of perceived auditory location. Psychon. Bull. Rev. 5, 482–489 10.3758/BF03208826

[B4] Carandini M. Heeger D. J. Movshon J. A. (1997). Linearity and normalization in simple cells of the macaque primary visual cortex. J. Neurosci. 17, 8621–8644 9334433 10.1523/JNEUROSCI.17-21-08621.1997 PMC6573724

[B5] Deneve S. Latham P. E. Pouget A. (1999). Reading population codes: a neural implementation of ideal observers. Nat. Neurosci. 2, 740–745 10.1038/11205 10412064

[B6] Doya K. Ishii S. Pought A. Rao R. P. N. (2007). Bayesian Brain: Probabilistic Approaches to Neural Coding. Cambridge, MA: MIT Press

[B7] Hairston W. D. Wallace M. T. Vaughan J. W. Stein B. E. Norris J. L. Schirillo J. A. (2003). Visual localization ability influences cross-modal bias. J. Cogn. Neurosci. 15, 20–29 10.1162/089892903321107792 12590840

[B8] Howard I. P. Templeton W. B. (1966). Human Spatial Orientation. New York, NY: Wiley

[B9] Hubel D. H. Wiesel T. N. (1962). Receptive fields, binocular interaction and functional architecture in the cat's visual cortex. J. Physiol. 160, 106–154 14449617 10.1113/jphysiol.1962.sp006837 PMC1359523

[B10] Körding K. P. Beierholm U. Ma W. J. Quartz S. Tenenbaum J. B. Shams L. (2007). Causal inference in multisensory perception. PLoS ONE 9:e943 10.1371/journal.pone.0000943 17895984 PMC1978520

[B11] Latham P. E. Deneve S. Pouget A. (2003). Optimal computation with attractor networks. J. Physiol. 97, 683–694 15242674 10.1016/j.jphysparis.2004.01.022

[B12] Lewald J. Ehrenstein W. H. Guski R. (2001). Spatio-temporal constraints for auditory–visual integration. Behav. Brain Res. 121, 69–79 10.1016/S0166-4328(00)00386-7 11275285

[B13] Ma W. J. Beck J. M. Latham P. E. Pouget A. (2006). Bayesian inference with probabilistic population codes. Nat. Neurosci. 9, 1432–1438 10.1038/nn1790 17057707

[B14] Marti D. Rinzel J. (2013). Dynamics of feature categorization. Neural Comput. 25, 1–45 10.1162/NECO_a_00383 23020108

[B15] Pavani F. Spence C. Driver J. (2000). Visual capture of touch: out-of-the-body experiences with rubber gloves. Psychol. Sci. 11, 353–359 10.1111/1467-9280.00270 11228904

[B16] Pouget A. Zhang K. Deneve S. Latham P. E. (1998). Statistically efficient estimation using population coding. Neural Comput. 10, 373–401 10.1162/089976698300017809 9472487

[B17] Sato Y. Toyoizumi T. Aihara K. (2007). Bayesian inference explains perception of unity and ventriloquism aftereffect: identification of common sources of audiovisual stimuli. Neural Comput. 19, 3335–3355 10.1162/neco.2007.19.12.3335 17970656

[B18] Shadlen M. N. Britten K. H. Newsome W. T. Movshon J. A. (1996). A computational analysis of the relationship between neuronal and behavioral responses to visual motion. J. Neurosci. 16, 1486–1510 10.1016/0959-4388(94)90059-0 8778300 PMC6578557

[B19] Shadlen M. N. Newsome W. T. (1994). Noise, neural codes and cortical organization. Curr. Opin. Neurobiol. 4, 569–579 7812147 10.1016/0959-4388(94)90059-0

[B20] Wallace M. T. Roberson G. E. Hairston W. D. Stein B. E. Vaughan J. W. Schirillo J. A. (2004). Unifying multisensory signals across time and space. Exp. Brain Res. 158, 252–258 10.1007/s00221-004-1899-9 15112119

